# High intensity interval training attenuate insulin resistance in diabetic rats accompanied by improvements in liver metabolism and spexin signaling

**DOI:** 10.1038/s41598-025-15432-8

**Published:** 2025-08-21

**Authors:** Kayvan Khoramipour, Najmeh Sadat Hosseini, Jennifer W. Hill, Karen Khoramipour, Kimya Khoramipour, Sergio Maroto Izquierdo, Simone Lista, Mona Saheli

**Affiliations:** 1 i+HeALTH Strategic Research Group, Department of Health Sciences, Miguel de Cervantes European University (UEMC), 47012 Valladolid, Spain; 2 Physiology Research Center, Institute of Neuropharmacology, Kerman University of Medical Sciences, Kerman, Iran; 3 Department of Physiology and Pharmacology, University of Toledo College of Medicine and Life Sciences, Toledo, OH 43614 USA; 4 Center for Diabetes and Endocrine Research, Toledo, OH 43614 USA; 5 Department of Sport Science, Faculty of Humanities and Social Sciences, Kurdistan University, Kurdistan, Iran; 6 Department of Nursing, Faculty of Nursing and Midwifery, Kurdistan University of Medical Sciences, Kurdistan, 66179-13446 Iran; 7 Endocrinology and Metabolism Research Center, Kerman University of Medical Sciences, Kerman, Iran

**Keywords:** HIIT, Gluconeogenesis, Lipolysis, Lipogenesis, Spexin, Physiology, Diseases, Endocrinology

## Abstract

Spexin (SPX), a recently identified adipokine, shows potential in enhancing insulin sensitivity, offering promising implications for metabolic health. This study aimed to investigate the effect of 8 weeks of high intensity interval training (HIIT) on insulin resistance through liver gluconeogenesis, lipolysis, inflammation, oxidative stress, and lipogenesis in Type 2 diabetic rats with a special focus on the role of SPX. In this study, 28 male Wistar rats were divided into four groups: Healthy Control (CON), Diabetes Control (T2D), HIIT, and Diabetes+HIIT (T2D+HIIT). After diabetes induction (high-fat diet+streptozotocin), the exercise groups underwent an 8-week HIIT protocol. HOMA-β, HOMA-IR, QUICKI, fasting blood sugar, insulin, and SPX were measured in liver and serum. The expression of Galanin/2, FOXO-1, PGC-1α, G6Pase, PEPCK, CPT1A, AMPK, PPARα, ACC, FAS, SREBP-1c, SIRT-1, and inflammatory/antioxidant markers were analyzed in the liver. Our results showed that HOMA-β, QUICKI, insulin, and serum level of SPX levels were higher in T2D+HIIT than T2D group. Similarly, SPX, GALR2, FOXO-1, PGC-1α, CPT1A, PPARα, AMPK, and SIRT-1 levels were higher in the livers of rats in the T2D+HIIT than T2D group. However, HOMA-IR, PEPCK, G6Pase, ACC, FAS, and SREBP-1c levels were lower in T2D+HIIT than T2D group. Also, HIIT+T2D reduced inflammation and increased antioxidants, indicating an improved overall health status of the liver. HIIT benefits diabetic liver with reducing gluconeogenesis, inflammation, oxidative stress, and lipogenesis while increasing lipolysis. These improvements coincided with elevated hepatic SPX signaling and are associated with reduced insulin resistance. These findings suggest a potential association between HIIT induced SPX signaling modulation in liver and improving insulin resistance, though further mechanistic studies are required to confirm the causality.

**Supplementary Information:**

The online version contains supplementary material available at 10.1038/s41598-025-15432-8.

## Introduction

Diabetes is a widespread metabolic disease defined by abnormal digestion of proteins, lipids, and carbohydrates as well as persistently elevated blood sugar levels^1,2^. By 2030, itis estimated that nearly 366 million people will be living with diabetes. Of these individuals, approximately 90% to 95% are expected to have type 2 diabetes (T2D), which is the most common form of the disease^3,4^.

Two key features of T2D are insulin resistance (IR) and elevated gluconeogenesis^5,6^.Studies have shown that adipokines, bioactive molecules (such as hormones and cytokines) secreted by adipose tissue, could affect these pathological process^5,6^. A 14-amino acid peptide, spexin (SPX) is the most recently discovered adipokine considered to be involved in a number of homeostatic processes, including metabolism, energy balance, and reproduction^6–8^. Its expression has been reported in the liver, adipose tissue, pancreas, kidneys, and skeletal muscle^9,10^. Previous clinical studies demonstrated that IR, obesity, and T2D are associated with low blood concentrations of SPX^11,12^. In addition, serum SPX concentrations negatively correlated with body mass index (BMI)^11,12^. Furthermore, SPX reduced rate of dyslipidemia, gluconeogenesis, lipogenesis, and hepatic fat accumulation but increased lipolysisin vitro^6,13^. Through its receptor (i.e. Galanin 2/3 (GAL2/3)), SPX stimulates upregulation of fatty acid translocase (FAT/CD36), carnitine palmitoyl transferase 1A (CPT1A), peroxisome proliferator-activated receptor alpha (PPAR-α), and peroxisome proliferator-activated receptor gamma coactivator 1-alpha (PGC-1α) in rat cardiomyocytes^7,14^.

Regular physical exercise was shown to increase SPX blood concentration^14,15^. Both resistance and aerobic training can stimulate SPX expression, reduce the levels of pro-inflammatory cytokines, such as interleukin-6 (IL-6) and tumor necrosis factor alpha (TNFα), and improve glucose uptake and insulin sensitivity in skeletal muscles^14,15^. Leciejewska et al.^16^ reported that exercise can increase not only SPX secretion but also GALR2 and GALR3 expression in peripheral tissues. In addition, it has been found that high intensity interval training (HIIT) is more effective in increasing the levels of SPX than moderate intensity aerobic training^17^. Therefore, the purpose of this study was to investigate the effect of HIIT on IR in T2D rats with special focus on the role of SPX in enhancing hepatic lipolysis and reducing gluconeogenesis and lipogenesis.

## Materials and methods

### Animal models

28 male Wistar rats weighing 130–160 g, with an average age of 7–8 weeks, were purchased from the animal farm of Kerman University of Medical Sciences. During the experiment, the animals were housed in a 12-h light/dark cycle at a constant temperature (22 ± 1/4 °C), and humidity (50 ± 4%), with access to food and water ad libitum. The animals were randomly divided into four groups (n = 7 in each group) following the adaptation period of one week: control (CON), type 2 diabetes (T2D), high intensity interval training (HIIT), and T2D + HIIT. Kerman University of Medical Sciences Experimental Animals Ethics Committee approved all procedures (Ethical code: IR.KMU.AEC.1404.005).

### Induction of diabetes

To induce T2D, we adapted the method introduced by Magalhaes et al.^18^, with modifications to made it more feasible in our lab. We used this method in our previous publications^19–21^. Animals in T2D and T2D+HIIT groups were fed a high-fat diet (HFD) for 2 months (60% fat, 20% carbohydrates, and 20% protein). Then, the animals were injected with streptozotocin (STZ) at a low dose (30 mg/kg). Blood glucose levels were measured with a glucometer 3 days after the injection, after 12 h of fasting. Animals with fasting blood glucose higher than 300 mg/dl were considered diabetic. Animals in CON and HIIT groups consumed a normal diet (20% fat, 60% carbohydrates, and 20% protein).

### Treadmill running protocol

After the induction of T2D, animals in HIIT and T2D+HIIT groups were acclimated to treadmill for 5 days (with speed of 8 m/min for 10 min). Then, we used stepwise testing to assess each rat’s maximum speed (Vmax). Starting with 6 m/min, the treadmill speed increased every 2 min until the rat reached exhaustion. Each rat’s final effort was taken as Vmax. Vmax was measured every two weeks. We have described the main training protocol in detail in previous publications^19,20,22–31^. Briefly, the protocol involved 4–10 intervals (2 min at high intensity and 1 min at low intensity) with 80–100% of Vmax (Table 1).

**Table 1:** Exercise protocol.

Week	Intervals	High intensity interval duration (min)	Low intensity interval duration (min)	High intensity interval velocity (%VMax)	Low intensity interval velocity (%VMax)	Total exercise time in a session (min)
1	4	2	1	80	50	12
2	4	2	1	85	50	12
3	6	2	1	85	50	18
4	6	2	1	90	50	18
5	8	2	1	90	50	24
6	8	2	1	95	50	24
7	10	2	1	95	50	30
8	10	2	1	100	50	30

While slope and frequency were the same for all 8 weeks, we applied additional load concept by increasing the intensity or duration of the protocol.

### Serum and tissue sampling

A combination of ketamine (80 mg/kg) and xylazine (10 mg/kg) was prepared fresh on the day of the procedure and administered via intraperitoneal injection in a total volume of 1 mL/kg body weight to euthanasia of the animals. Then, blood samples were taken from the animals’ heart. The whole liver tissue was then carefully removed, immediately frozen in liquid nitrogen, and stored at 80 °C for further analysis.

### ELISA

Inflammatory and anti-inflammatory cytokines, namely TNF-α and interleukin-10 (IL-10) (Karmania Pars Gene Company, Iran), insulin serum concentration (The ALPCO Company, United States), Spexin in serum and liver (PHOENIX PHARMACEUTICALS, INC, United States), ALT (Karmania Pars Gene Company, Iran), and AST (Karmania Pars Gene Company, Iran) were all measured according to the instructions of their respective ELISA kits^32,33^.

### Measurement of oxidative stress indices

The measurement of glutathione peroxidase (GPX) was based on the ability of glutathione peroxidase to oxidize glutathione (GSH) to oxidized glutathione (GSSH). Glutathione reductase converts GSSH to GSH using nicotinamide dinucleotide phosphate (NADPH). The decrease in NADPH levels, measured at 340 nm, indicated GPX activity, which was measured using a kit (Behboud Tahghigh Kerman Company, Iran). The activity of SOD and CAT was indirectly measured using a colorimetric method inhibiting pyrogallol oxidation, according to the kits instruction(Behboud Tahghigh Kerman Company, Iran).

### Western blotting

All antibodies used in the study were purchased from Santa Cruz Biotechnology, Inc, U.S.. The expression of galanin (GAL) (sc-16219), forkhead box protein O1 (FOXO-1) (sc-374427), peroxisome coactivator 1 alpha (PGC-1α) (sc-544812), carnitine palmitoyl transferase 1A (CPT1A) (sc-514555), Peroxisome proliferator-activated receptor α (PPARα) (sc-398394), Phosphoenolpyruvate carboxykinase (PEPCK) (sc-271029), Sirtuin 1 (SIRT-1) (sc-523698), Glucose 6-phosphatase (G6Pase) (sc-25840), Acetyl-CoA carboxylase (ACC) (sc-140258), Fas Fatty acid synthase (FAS) (sc-263879), AMP-activated protein kinase (AMPK) (sc-104369), and Sterol regulatory element-binding protein 1 (SREBP-1c) (sc-323698) were assessed using the western blotting technique. Western blot protocol has been described in detail elsewhere^18,34^. Firstly, liver samples were lysed using lysis buffer (150 mM sodium chloride, 1% Triton X-100, 0.2% deoxycholate sodium, 0.1% SDS, 50 mM Tris, pH 8.0). Using an Eppendorf 5415R centrifuge, the samples were centrifuged at 12,000 rpm for 10 min at 4 °C. The supernatant was collected. A Bradford assay determined the protein concentrations in samples stored at 80 °C. After separation by SDS-PAGE, the proteins were transferred to a PVDF membrane at 120 mV for 1.5 h. Incubation with specific primary antibodies was conducted for 18 h after blocking the membranes with 5% skim milk for 1 h at room temperature. After washing with TBS-T, the membranes were incubated with secondary antibodies conjugated with horseradish peroxidase. Using ECL reagents, the proteins of interest were visualized. Finally, ImageJ software converted qualitative measurements to quantitative information.

### Calculation of insulin resistance/sensitivity indices

The Homeostasis Model Assessment (HOMA) was utilized to evaluate insulin resistance (HOMA-IR) and β-cell function (HOMA-β). HOMA-IR and HOMA-β scores were computed using the following formulas: HOMA-IR = [(fasting glucose (mmol/l) × fasting insulin (μU/ml))/22.5]. HOMA β-cell = [(20 × fasting insulin (μU/ml))/(fasting glucose (mmol/l) − 3.5)^35^. The Quantitative Assessment of Insulin Sensitivity Index (QUICKI) was also employed due to its strong correlation with glucose clamp determination of insulin sensitivity. QUICKI was calculated using the formula of Katz and colleagues: (i.e., 1/[log (fasting insulin in μU/ml) + log (fasting glucose in mg/dl)])^36^.

### Statistical analysis

Results are shown as the mean ± standard deviation (SD). Data normality was assessed with the Kolmogorov–Smirnov test. To compare variables between groups, we employed a Two-Way ANOVA followed by a Tukey post-hoc test. All statistical analyses were conducted using GraphPad Prism version 9.0. A *p*-value less than 0.05 was considered statistically significant.

## Results

### Body weight and blood glucose concentrations

**Fig. 1:**
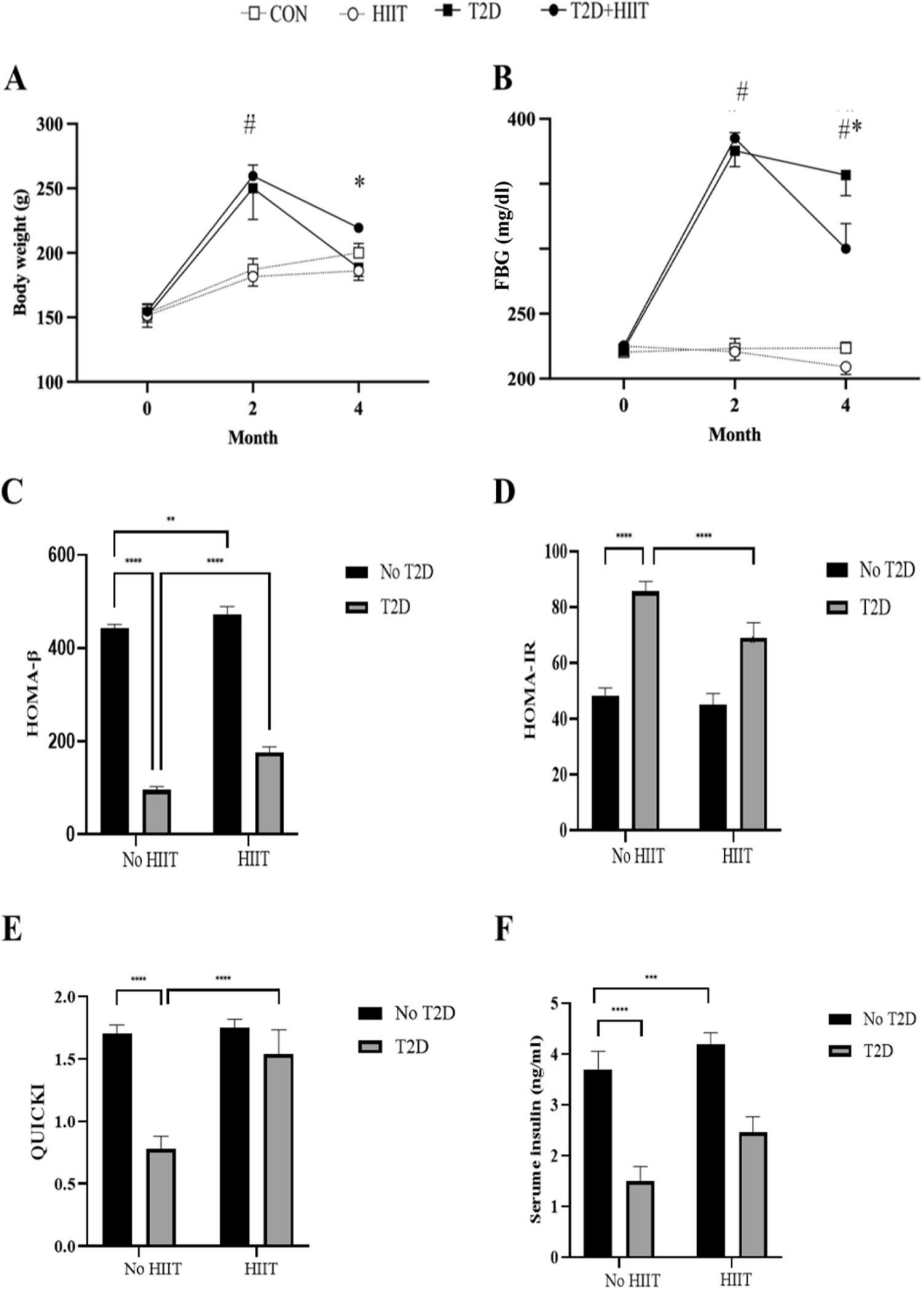
Body weight (**A**) and fasting blood glucose (FBG) (**B**) before starting the intervention (month 0), after diabetes induction (month 2), and 48 h after the last training session (month 4) in all groups. The homeostatic model assessment (HOMA)-β (**C**), HOMA of insulin resistance (HOMA-IR) (**D**), Quantitative insulin sensitivity check index (QUICKI) (**E**), serum insulin (**F**) (Mean ± SD, n = 7 in each group). Abbreviations: CON: control, T2D: type 2 diabetic , HIIT: high intensity interval training. ^#^(*P* < 0.0001) significant difference between T2D and T2D + HIIT with other groups, *(*P* < 0.01) significant difference between T2D and T2D + HIIT. ****(*P* < 0.0001), ***(*P* < 0.001), **(*P* < 0.05).

The data indicated a significant increase in body weight in both T2D and T2D+HIIT compared to CON and HIIT groups after diabetes induction (month 2) (*P* < 0.0001). However, month 4 body weight was reduced in the T2D and T2D+HIIT compared to CON and HIIT groups (*P* < 0.01), with a greater decrease in the T2D group (*P* < 0.0001) (Fig. 1A). Fasting blood glucose (FBG) was significantly increased after diabetes induction in both T2D and T2D+HIIT groups compared to the baseline (*P* < 0.0001). HIIT led to reduce FBG values in T2D+HIITcompared to the T2D group (*P* < 0.0001) (Fig. 1B).

### Insulin resistance/sensitivity indexes, and serum insulin concentrations

HOMA-β index was higher in the HIIT group (*P* < 0.0001, 6.74% higher) and lower in the T2D one (*P* < 0.0001, 82.47% lower) compared to the CON group. Additionally, the interaction between T2D and HIIT was significant (*P* < 0.0001, 55.68% higher in T2D+HIIT compared with T2D) (Fig. 1C).

HOMA-IR index was significantly higher in T2D compared to the CON group (*P* < 0.0001, 43.46% higher). There were no significant changes in HOMA-IR levels between CON and HIIT groups. However, HOMA-IR index was lower in the T2D+HIIT group compared to the T2D group (interaction effect) (*P* < 0.001, 19.39% lower) (Fig. 1D).

QUICKI index were significantly lower in the T2D group compared to the CON one, (*P* < 0.0001, 118.46% lower). Furthermore, significant interaction between T2D and HIIT groups was found (*P* < 0.0001, 97.18% higher in T2D+HIIT compared to T2D group) (Fig. 1E).

Serum insulin concentrations were significantly lower in the T2D group compared to the CON (*P* < 0.0001, 213.79% lower). and the T2D+HIIT (*P* < 0.0001, 64.66% lower) groups (Fig. 1F).

### GAL-R2 expression levels in the liver and SPX expression levels in liver and serum concentrations

**Fig. 2:**
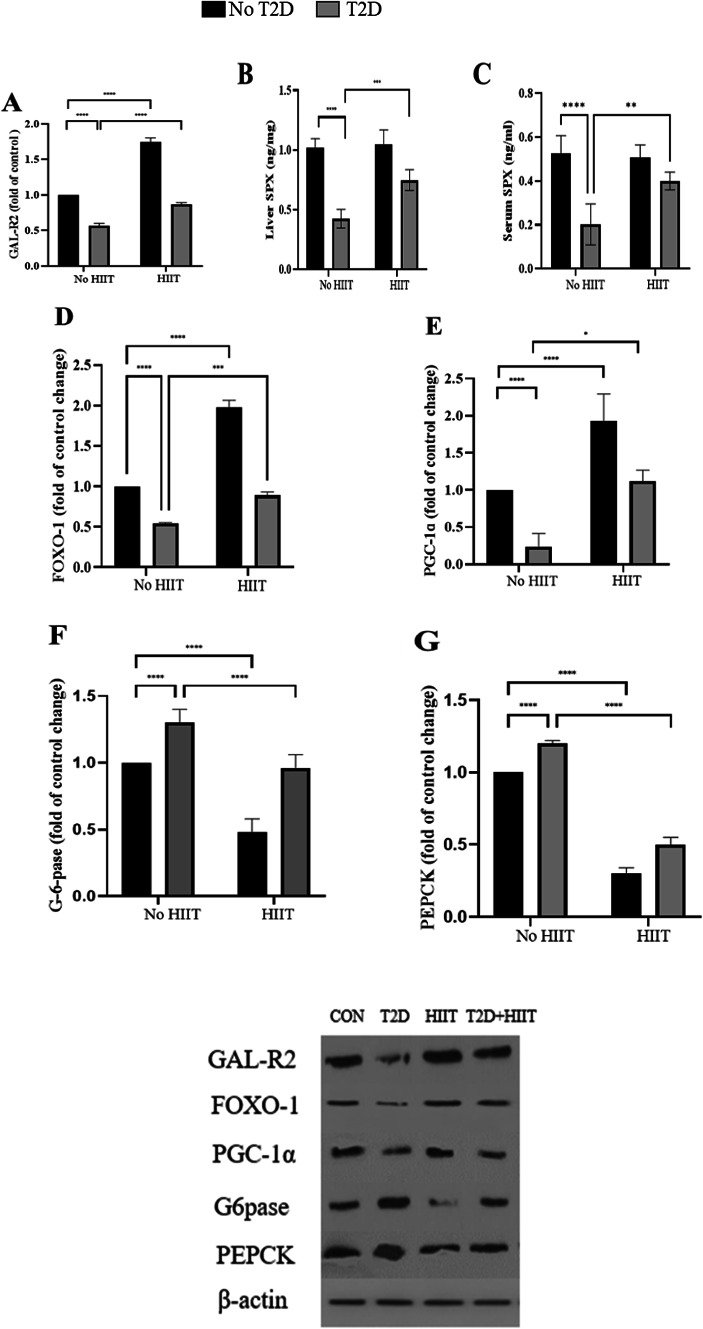
GAL-R2 (**A**), liver SPX (**B**), serum SPX (**C**) FOXO-1 (**D**), PGC-1α (**E**), G6Pase (**F**), and PEPCK (**G**) expression in the liver (mean ± SD, n = 7 in each group). *GAL-R2* Galanin receptor 2, *SPX* spexin, *FOXO-1* Forkhead box protein O, *PGC-1α* peroxisome proliferator-activated receptor gamma coactivator (PGC)-1alpha, *G6Pase* glucose 6-phosphatase, *PEPCK* phosphoenolpyruvate carboxykinase, *CON* control, *T2D* Type 2 diabetes, *HIIT* high intensity interval training. ****(*P* < 0.0001), ***(*P* < 0.001), **(*P* < 0.01), and *(*P* < 0.05).

HIIT significantly increased GAL-R2 expression levels in the liver (*P* < 0.0001, 70% increase). Conversely, T2D was associated with a significant decrease in GAL-R2 liver expression (*P* < 0.0001, 43.11% decrease). GAL-R2 levels were significantly higher in the T2D+HIIT group compared to the T2D group (*P* < 0.001, 32.48% higher) (Fig. 2) (All western blot images have been provided in Supplementary material).

Both SPX liver expression levels (*P* < 0.0001, 60.23% lower) and serum concentrations (*P* < 0.0001, 59.41% lower) were significantly lower in the T2D group compared to the CON one. However, the T2D+HIIT (interaction effect) group showed higher SPX values in both serum (*P* < 0.001, 98.02% higher) and liver (*P* < 0.001, 76.42 higher) compared to the T2D group (Fig. 2B,C).

### FOXO-1, PGC-1α, G6Pase, and PEPCK expression levels in the liver

HIIT significantly increasedFOXO-1 expression levels compared to CON group in the liver (*P* < 0.0001, 97.32% decrease). In contrast, the T2D group exhibited a substantial decrease in expression levels ofFOXO-1 compared to CON group in the liver (*P* < 0.0001, 48.62% decrease). Moreover, this group presented lower levels of FOXO-1 compared to the T2D+HIIT group (*P* < 0.0001, 35.21% lower) (Fig. 2D).

HIIT significantly increased PGC-1α expression levels compared to CON group in the liver (*P* < 0.0001, 96.71 increase). Conversely, T2D was associated with a remarkable decrease in PGC-1α levels (*P* < 0.0001, 72.14% decrease). Furthermore, PGC-1α levels were significantly higher in the T2D+HIIT group compared to the T2D one (*P* < 0.05, 79.23% higher) (Fig. 2E).

G6Pase expression levels were lower in the HIIT group (*P* < 0.0001, 55.36% lower) and higher in the T2D group (*P* < 0.0001, 23.63% higher) compared to the CON one. Furthermore, significant interaction effect was observed for G6Pase (*P* < 0.0001, 32.21% lower in T2D+HIIT compared with T2D).Furthermore, PEPCK showed higher levels in the T2D group compared to the CON (*P* < 0.0001, 17.32% higher) and the T2D+HIIT (*P* < 0.0001, 60.23% change) ones. HIIT also decreased PEPCK compared with CON group (*P* < 0.0001, 78.23% decrease) (Fig. 2F,G) (All western blot images have been provided in Supplementary material).

### CPT1A, PPARα and SIRT1 expression levels in the liver

**Fig. 3:**
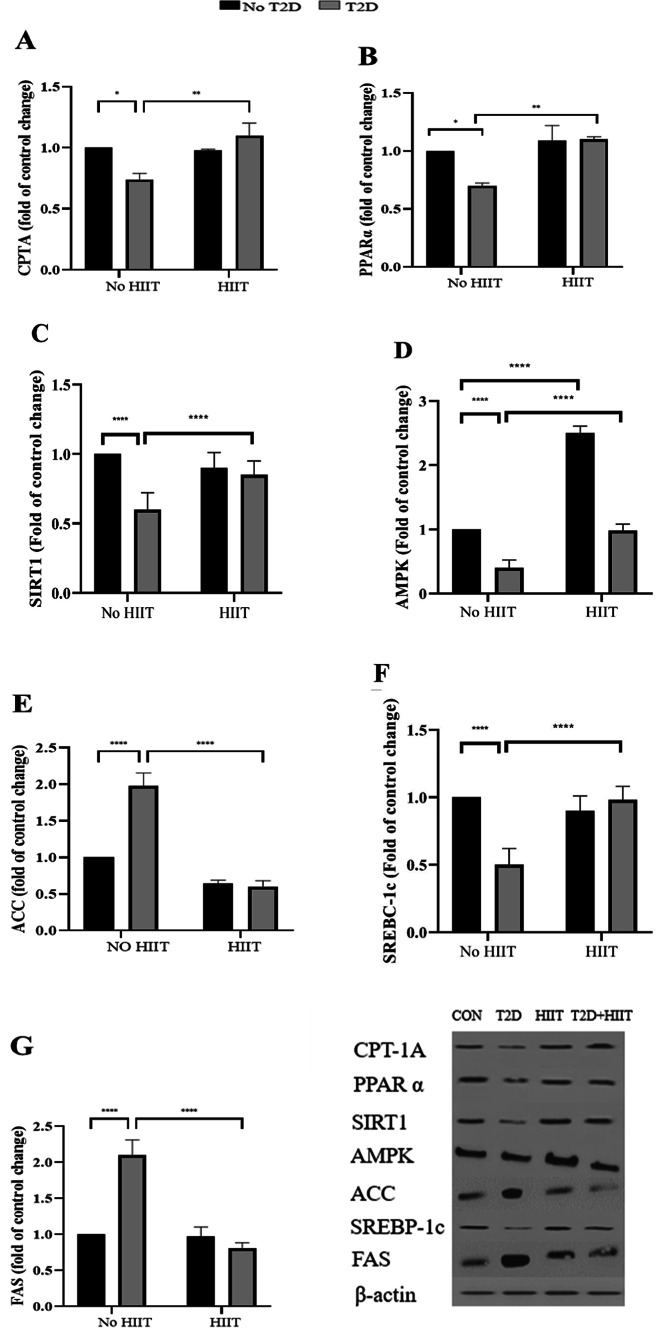
CPT1A (**A**), PPARα (**B**), and SIRT-1 (**C**) AMPK (**D**), SREBP-1c (**E**), ACC (**F**) and FAS (**G**) expression in the liver (Mean ± SD, n = 7 in each group). *CPTIA* carnitine palmitoyl transferase IA, *PPARα* peroxisome proliferator-activated receptor α, *AMPK* AMP-activated protein kinase, *SREBP-1c* sterol regulatory element-binding protein 1, *ACC* Acetyl-CoA carboxylase, *FAS* fatty acid synthase, *CON* control, *T2D* Type 2 diabetes, *HIIT* high intensity interval training. ****(*P* < 0.0001), **(*P* < 0.01).

Both CPT1A and PPARα expression levels were significantly lower in the T2D group compared to the CON one (*P* < 0.05, 20.04% lower and *P* < 0.05, 27.87% lower, respectively). The T2D + HIIT group exhibited significantly higher levels of both CPT1A (*P* < 0.001, 26.14% higher) and PPARα (*P* < 0.01, 25.25% higher) compared to the T2D group (Fig. 3A,B) (All western blot images have been provided in Supplementary material).

Additionally, SIRT-1 expression levels were significantly lower in the T2D group compared to the CON (*P* < 0.05, 39.32% lower) and the T2D+HIIT (*P* < 0.0001, 32.62% lower) ones (Fig. 3C).

### AMPK, SREBP-1c, ACC, and FAS expression levels in liver

HIIT led to a significant increase in AMPK expression levels compared to CON group (*P* < 0.0001, 156.57% increase). Conversely, the T2D group was associated with a significant decrease in AMPK levels in the liver (*P* < 0.0001, 60.79% decrease). Furthermore, AMPK levels were significantly higher in the T2D+HIIT group than in the T2D one (*P* < 0.0001, 60.56% higher). (Fig. 3D).

Rats ACC levels were significantly higher in the T2D group compared to the CON (*P* < 0.0001, 97.36% change). and T2D+HIIT ones (*P* < 0.0001, − 387.36% higher) (Fig. 3E). Rats with T2D exhibited lower SREBP-1c levels compared to both the CON group (*P* < 0.01, 49.58% lower) and the T2D+HIIT group (*P* < 0. 01, 39.86% lower) (Fig. 3F).

In addition, FAS levels were higher in the T2D group (*P* < 0.0020, 112.69% higher) compared to the CON and the T2D+HIIT ones (*P* < 0.0001, 125.98% higher) (Fig. 3G) (All western blot images have been provided in Supplementary material).

### TNFα and IL-10 expression levels in the liver

**Fig. 4:**
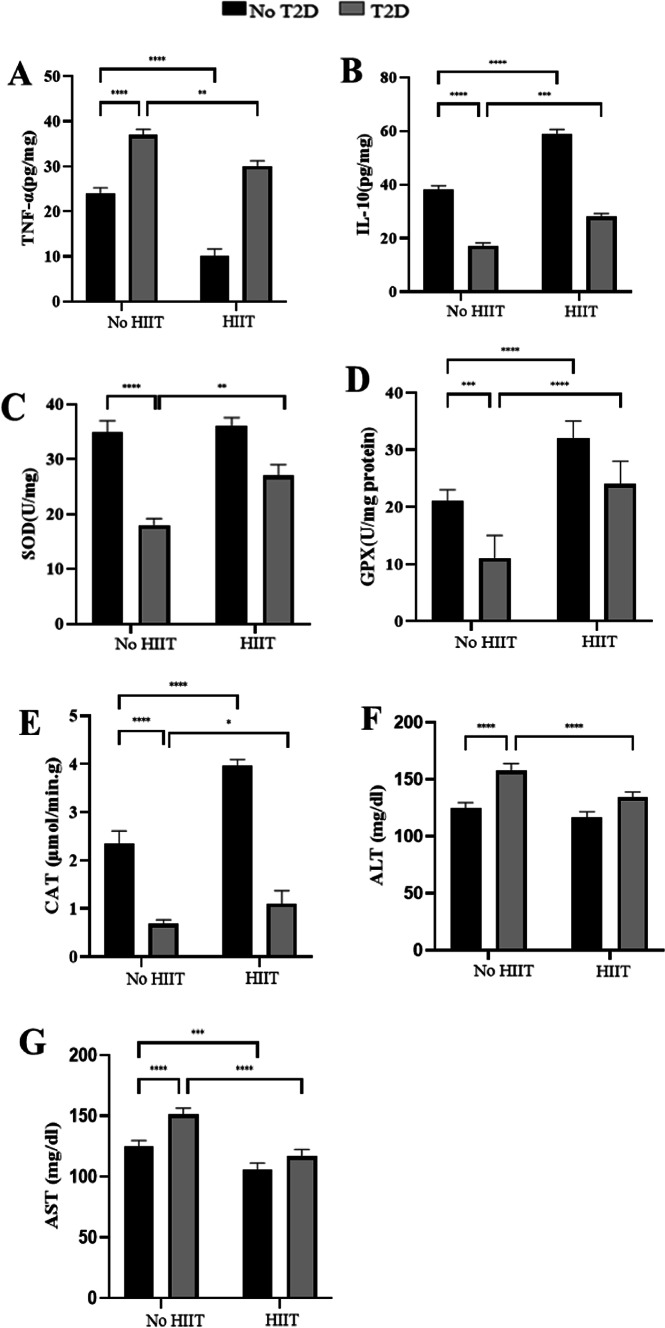
TNFα (**A**) and IL-10 (**B**), SOD (**C**), GPX (**D**) and CAT (**E**) levels in the liver, and serum levels of ATL (**F**) and AST (**G**) (Mean ± SD, n = 7 in each group). *TNFα* tumor necrosis factor alpha, *IL-10* interleukin-10, *SOD* superoxide dismutase, *GPX* glutathione peroxidase, *CAT* catalase, *ALT* alanine aminotransferase, *AST* aspartate aminotransferase, *CON* control, *T2D* Type 2 diabetes, *HIIT* high intensity interval control. ^****^(*P* < 0.0001), ^***^(*P* < 0.001), ^**^(*P* < 0.01), ^*^(*P* < 0.05).

TNFα expression levels in the liver were significantly lower in the HIIT group (*P* < 0.0001, − 59.39% lower) and higher in the T2D group (*P* < 0.0001, − 34.26% higher) compared to the CON one. Furthermore, TNFα levels were significantly lower in the T2D+HIIT group than the T2D one (*P* < 0.01, − 18.22% lower) (Fig. 4A).

HIIT was associated with increased IL-10 levels (*P* < 0.0001, 33.75% increase) andT2D was associated with decreased IL-10 levels compared to CON group (*P* < 0.0001, 59.62% change). Furthermore, IL-10 levels were significantly higher in the T2D+HIIT group compared to T2D one (*P* < 0.01, 32.31% change) (Fig. 4B).

### SOD, GPX, and CAT expression levels in in the liver

SOD expression levels in the liver were significantly lower in the T2D group compared to the CON (*P* < 0.0001, 46.36% lower) and the T2D+HIIT (*P* > 0.0001, 40.74% lower) groups (Fig. 4C).

Also, GPX levels were significantly different between CON and T2D groups (*P* < 0.0001, 38.10% lower in T2D).Furthermore, the HIIT group showed higher levels of GPX than the CON group (*P* < 0.0001, 31.03% higher). Moreover, there was a significant difference between T2D and T2D+HIIT groups (*P* < 0.01, 61.90% lower in T2D compared to HIIT+T2D) (Fig. 4D).

In addition, the HIIT (*P* < 0.0001, 169.23 higher) and the T2D (*P* < 0.0001, 65.12% lower) groups showed higher and lower CAT levels compared to the CON group, respectively. Finally, CAT levels were significantly higher in the T2D+HIIT group than in the T2D one (*P* < 0.0001, 61.76% higher) (Fig. 4E).

### ALT and AST concentrations in serum

ALT concentrations in serum were significantly higher in the T2D group compared to the CON (*P* < 0.0001, 21.04% higher) and the T2D+HIIT groups (*P* < 0.01, 14.83% higher) (Fig. 4F).

Furthermore, HIIT (*P* < 0.001, 15.41% lower) and the T2D (*P* < 0.0001, 17.70% higher) groups showed lower and higher serum concentrations of AST compared to the CON group, respectively. Moreover, serum AST concentrations were significantly lower in the T2D+HIIT group compared to the T2D one (*P* < 0.0001, 22.72% lower) (Fig. 4G).

## Discussion

The aim of the study was to investigate the effect of HIIT on IR through hepatic lipolysis, gluconeogenesis, and lipogenesis with a particular focus on adipokine SPX in a T2D rat model.

Our findings disclosed that 8-week HIIT protocol upgraded significantly the expression levels of SPX and GAL-R2 in liver of diabetic rats. Furthermore, an increase in FOXO1 and PGC1 as well as a decrease in G6Pase, suggested a decline in gluconeogenesis. Promotion of lipolysis confirmed by high expression of CPT1A, PPARY, and SIRT-1 in T2D+HIIT compared to T2D group. Also, elevation of AMPK and SREBP and reduction of ACC and FAS verified reduced lipogenesis. Exercise training was found to raise SPX serum concentrations and its expression levels in the adipose tissue of obese mice^14^ and humans^37^. Moreover, moderate and constant intensity exercise has demonstrated the ability to enhance adipose tissue SPX expression and secretion, resulting in reduced obesity-related glucose intolerance and IR^14^. A 12-week combined training can effectively improve SPX levels, lipid accumulation products, visceral adiposity index, and body composition in adults with T2D^15^. Likewise, chronic treatment with SPX improved glucose tolerance and decreased IR in obese and T2D rats^38,39^. Our results showed that SPX serum concentrations and liver expression levels decreased in T2D, which was associated with higher HOMA-IR and lower HOMA-β and QUICKI values. These data showed higher IR values in T2D group which can also affect the lower weight of this group compared to the CON one in month 4. In the absence of insulin, the body’s inability to effectively use glucose for energy leads to the breakdown of fat and muscle tissue, while excess glucose is excreted in urine (glycosuria) causing calorie deficit, and the metabolic shift to alternative energy sources further increases energy demand—all of which contribute to significant weight loss in uncontrolled diabetes^40,41^. However, 8 weeks of HIIT treatment could increase both the serum and liver level of SPX, as well GAL-R2 liver expression.

Likewise, these changes were associated with HOMA-β and QUICKI values improvement. These data suggested a possible role for SPX in the improvement of IR. However, mechanistic studies should examine this hypothesis. Recent evidence showed that SPX treatment diminished glucose-induced insulin release in isolated pig islets^42^. Also, glucose tolerance improvement and reduction of IR and hepatic lipids was observed after chronic treatment with SPX in both obese and T2D rats^38,39^. Our study highlights the potential role of SPX in improving IR in rodents. This modification was possibly mediated by binding of SPX to GALR2 and, finally, by the promotion of FOXO1/PGC-1α and suppression of G6Pase and PEPCK. However, while we did not directly investigate SPX binding to GALR2, previous studies reported the interaction of SPX with GALR2^43^ and its modulatory role on metabolic pathways associated with gluconeogenesis regulation, including insulin signaling and oxidative stress^18^. The regulation of both PEPCK and G6Pase genes at the transcriptional level involves the crosstalk between a network of transcription factors^44^. Indeed, transcription factors, such as FOXO1 and PGC-1α, stimulated the PEPCK promoter^9^. Previous studies confirmed the key role of PGC-1α in regulating glucose and lipid metabolism^45^. Thus, reduction of PGC-1α function is involved in impaired insulin signaling, reduced glucose uptake, and increased hepatic glucose production. These effects were followed by higher blood glucose concentrations and contributed to diabetes development^46^.

Moreover, PGC-1α signaling reduction is engaged in disrupted lipid metabolism, which is characterized by elevated lipogenesis and reduced fatty acid oxidation. Recent evidence showed that liver adenovirus-mediated overexpression of PGC-1α leads to significantly enhancement of glucose production in rat^47^. Notably, PGC-1α regulates transcription genes, such as PEPCK, G6Pase and FOXO1, where FOXO1 is a transcription factor modulating the gluconeogenic enzyme expression^48^.

Our data demonstrated that exercise raised SPX, FOXO1 and PGC-1α expression levels in the liver. These findings suggest that a partnership between PGC-1α and FOXO1 could possibly represent a mechanism monitoring gluconeogenesis. Given that SPX knockdown leads to upregulation of PEPCK and G6Pase, causing gluconeogenesis in HepG2 cells^6^, SPX maight be involved in the inhibition of G6Pase and PEPCK. Consequently, the binding of SPX to GALR2 likely could possibly promoted the FOXO1/PGC-1α expression. Previous studies showed that SPX knockdown results in increased levels of PEPCK and G6Pase, and, eventually, induction of gluconeogenesis in HepG2 cells^6^. SPX could also promotes lipolysis by enhancing the expression levels of CPT1A, PPARα, and PGC-1α^49^. Wang et al. reported that the stimulation of fatty acids metabolism and the breakdown of lipoproteins induced by SPX administration They explained that SPX increased FAT/CD36, CPT1A, PPARα, SIRT1, and PGC-1α expression^10^.

Furthermore, our findings revealed that the increased values of SPX following exercise was associated with an increase in AMPK expression. The latter downregulates the expression of SREBP-1c through the mTOR/AKT pathway inhibition. This might suggests a potential mechanism by which SPX could inhibit lipogenesis^50^. Also, the synthesis of malonyl-CoA from fatty acids decreased by SPX, indicates that SPX could possibly reduced the ACC function, a key enzyme responsible for the synthesis of malonyl-CoA from fatty acids, through AMPK activation^51^. Therefore, the synthesis of malonyl-CoA, a relevant component of lipid metabolism, decreased through the suppression of ACC by AMPK. Two key regulators of lipogenesis are FAS and ACC: the suppression of ACC induced by AMPK regulates SREBP-1c, a major transcription factor for the modulation of FAS and ACC^52^.

Recent evidence showed that AMPK activation can restrict inflammatory responses in different types of cells and tissues^53,54^. Inflammatory responses inhibition is mediated by several transcription factors, including SIRT1, PGC-1α, and FOXOs, that are downstream targets of AMPK signaling. Thus, AMPK signaling plays a role in inflammation reduction. Furthermore, the increase of antioxidant enzymes (i.e., SOD, GPX and CAT) defines the possible reason for reducing inflammatory responses in our study^53^.

This study presents some limitations. First, while food intake was not specifically monitored during the study, it is important to consider that variations in food consumption could influence metabolic outcomes. Moreover, body composition data was not assessed in this study. However, changes in body composition could significantly impact metabolic health and the response to HIIT. Future research should incorporate food monitoring and body composition measurements. Second, male rats were used in this study to reduce variability and control for sex-specific hormonal influences on metabolic outcomes. Previous research showed that male and female rats can respond differently to metabolic interventions due to hormonal differences. By using only males, we aimed to obtain more consistent and interpretable results. Future studies should include both sexes to explore potential sex-specific effects. Third, in future studies, GalR2 and GalR3 can be blocked by a selective antagonist, such as M871 or SNAP 37,889, to confirm the cause-and-effect relationship.

## Conclusions

In summary, our findings demonstrate that an 8-week HIIT protocol in type 2 diabetic rats was associated with beneficial changes in hepatic metabolism and inflammation. Specifically, HIIT reduced markers of lipogenesis and gluconeogenesis, increased indicators of lipolysis, and improved antioxidant status, while also upregulating SPX signaling and related proteins in the liver. These results were accompanied by improvements in insulin sensitivity and liver function markers.

While our data suggest that HIIT may exert its positive effects in part through modulation of SPX and its downstream pathways, the precise mechanistic links—particularly regarding the role of GALR2 and the FOXO1/PGC-1α axis—remain to be fully elucidated. Our study did not directly establish causality or the specific molecular mechanisms underlying these associations. Therefore, further targeted studies are warranted to clarify whether SPX acts via GALR2 to influence insulin resistance and hepatic metabolism, and to explore the involvement of the FOXO1/PGC-1α pathway in these processes.

## Supplementary Information

Below is the link to the electronic supplementary material.

Supplementary Material 1

## Data Availability

The datasets used and/or analysed during the current study are available from the corresponding author on reasonable request.
