# Supplementary figures and images for: High intensity interval training attenuate insulin resistance in diabetic rats accompanied by improvements in liver metabolism and spexin signaling
Source: Sci Rep. 2025 Aug 21;15:30682. doi: 10.1038/s41598-025-15432-8 (PMC12370946; doi:10.1038/s41598-025-15432-8)

GALR2

| 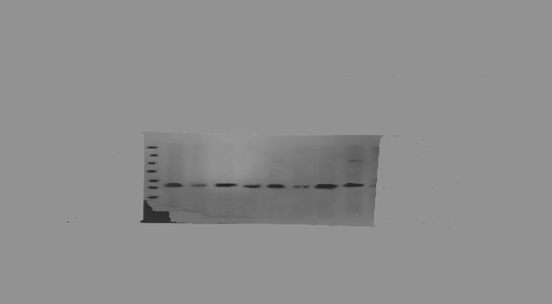 | 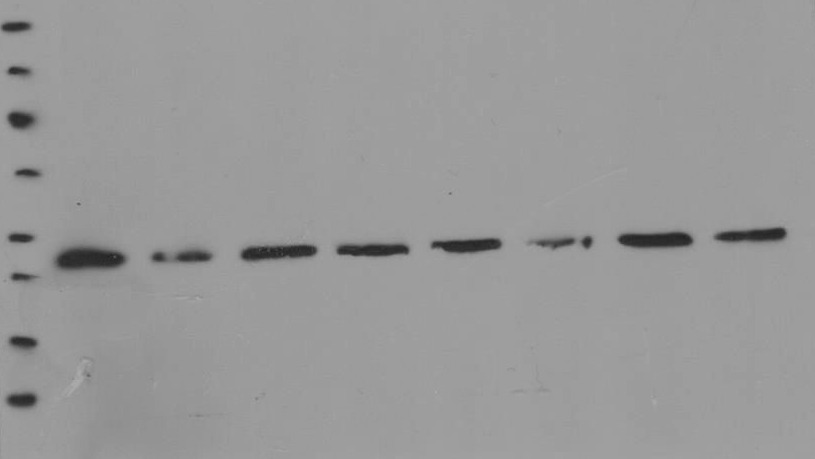 |
| --- | --- |
| 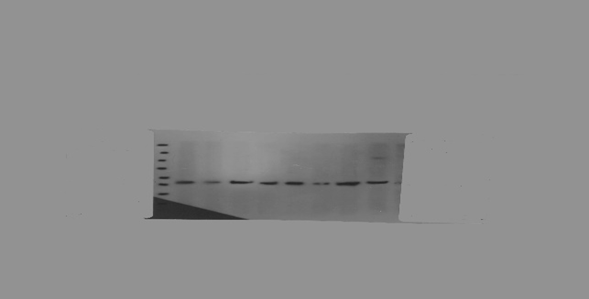 | |

FOXO-1

| 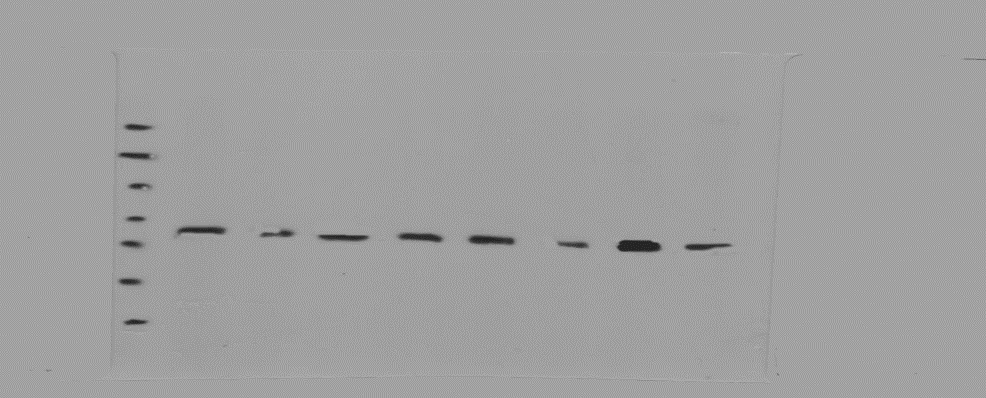 | 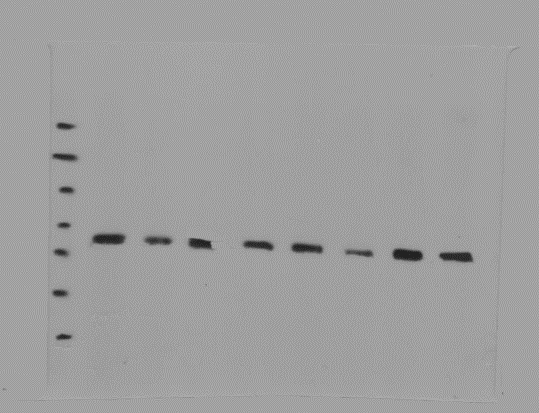 |
| --- | --- |
| 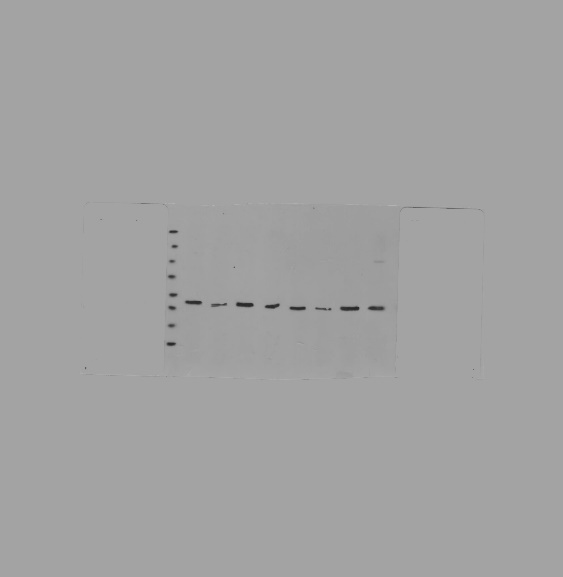 | |

PGC1α

| 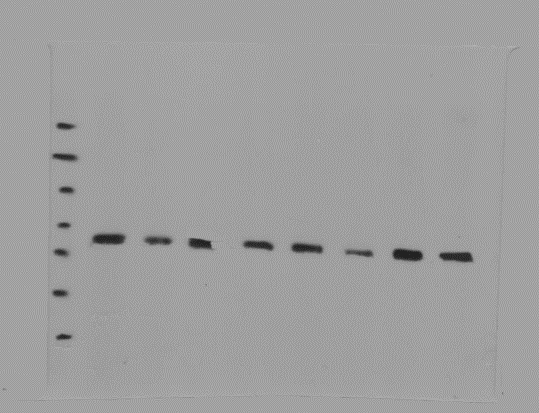 | 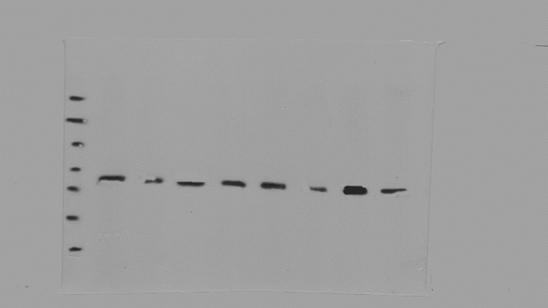 |
| --- | --- |
| 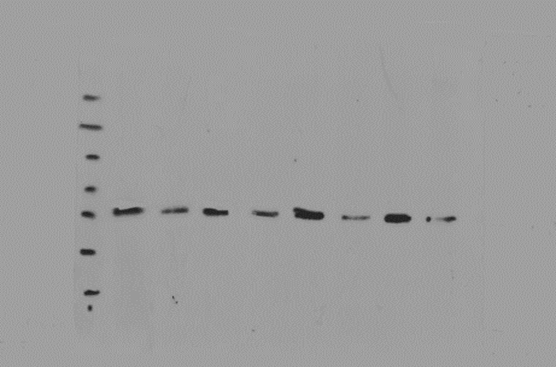 | |

G6Pase

| 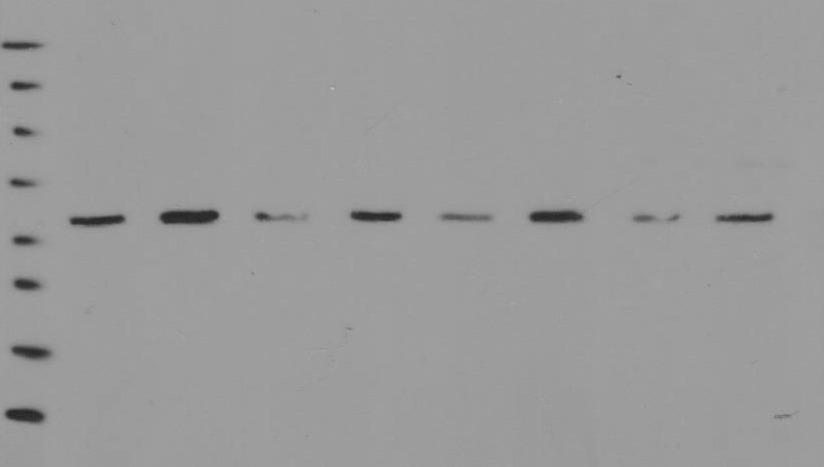 | 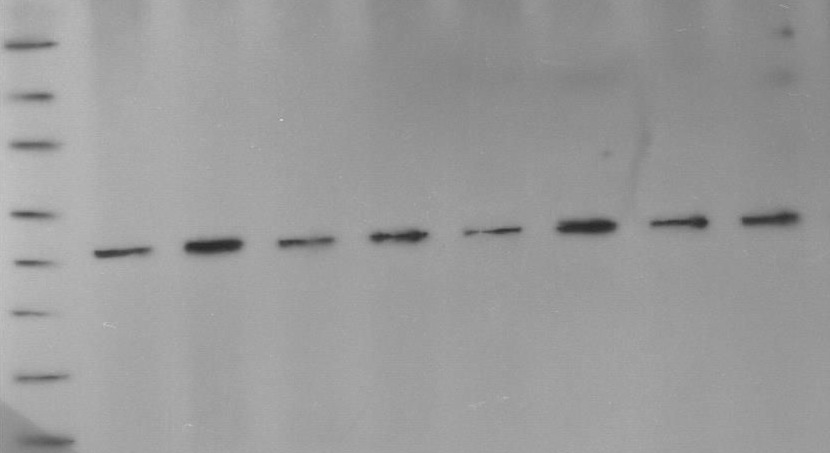 |
| --- | --- |
| 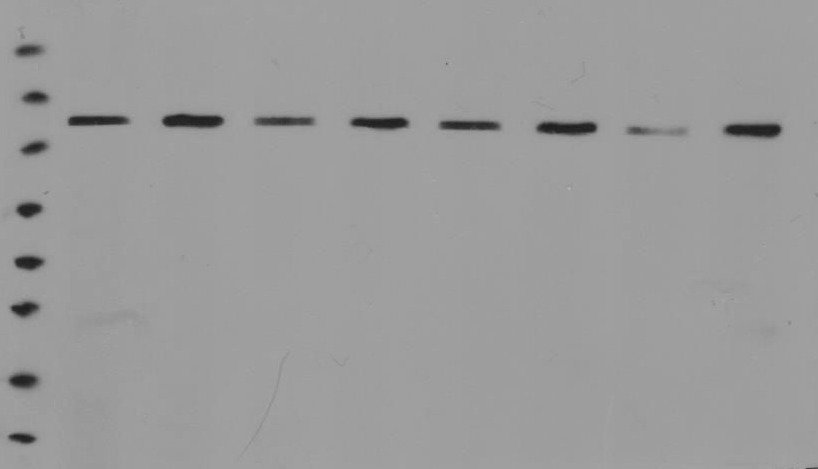 | |

PEPKC

| 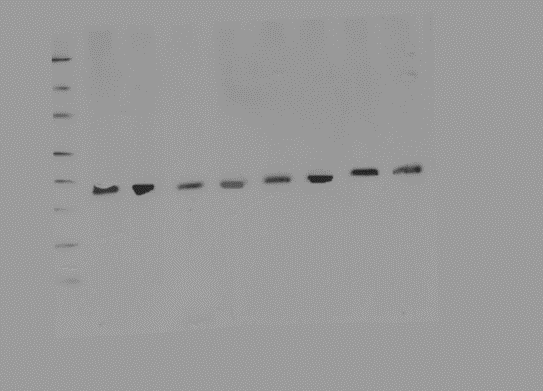 | 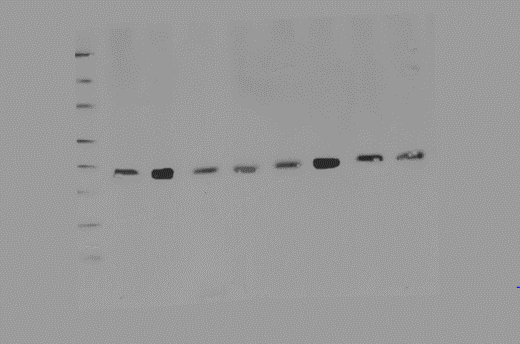 |
| --- | --- |
| 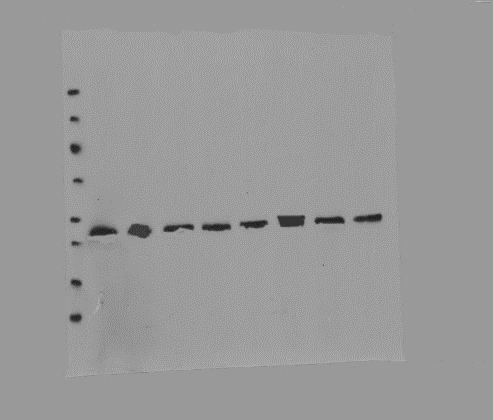 | |

CAPT1A

| 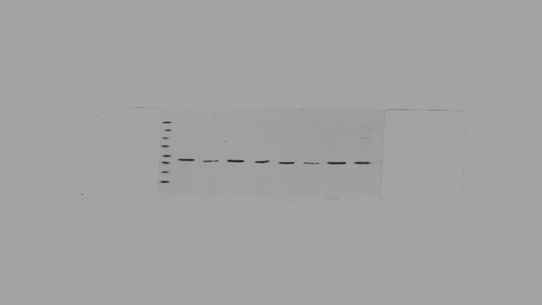 | 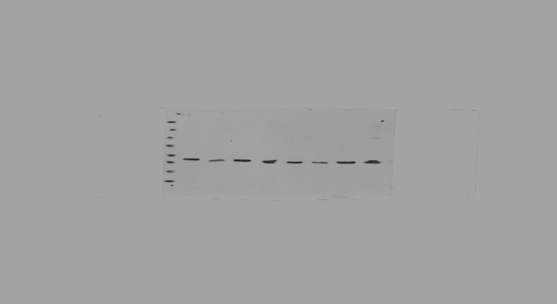 |
| --- | --- |
| 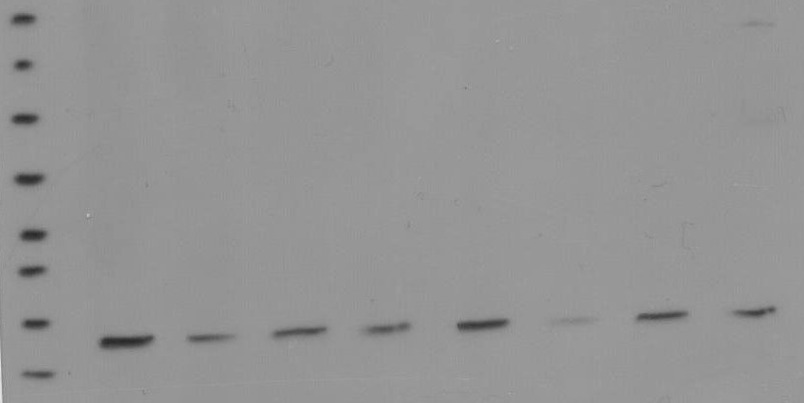 | |

PPARα

| 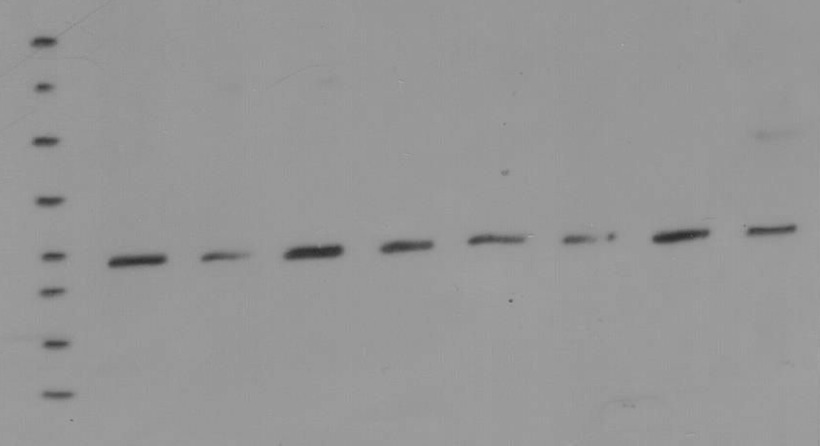 | 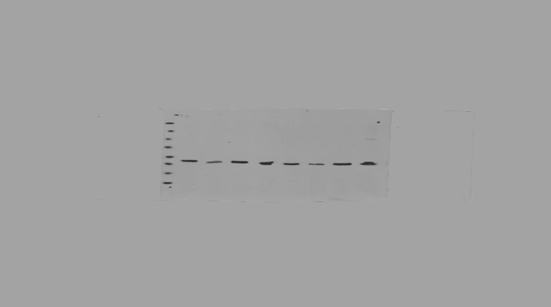 |
| --- | --- |
| 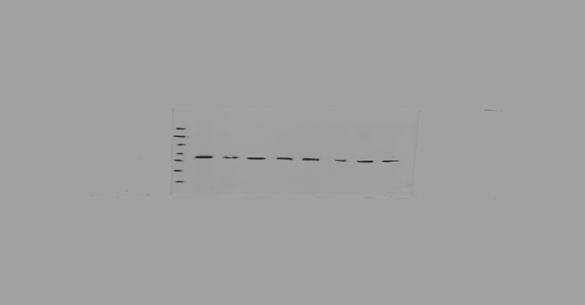 | |

SIRT1

| 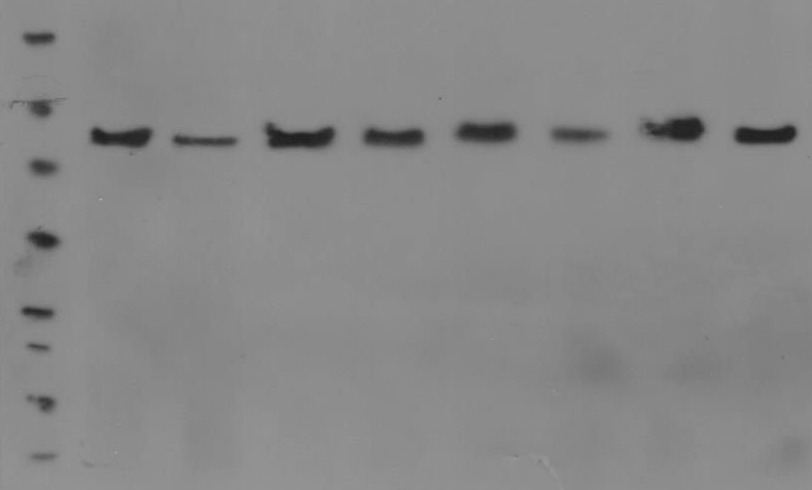 | 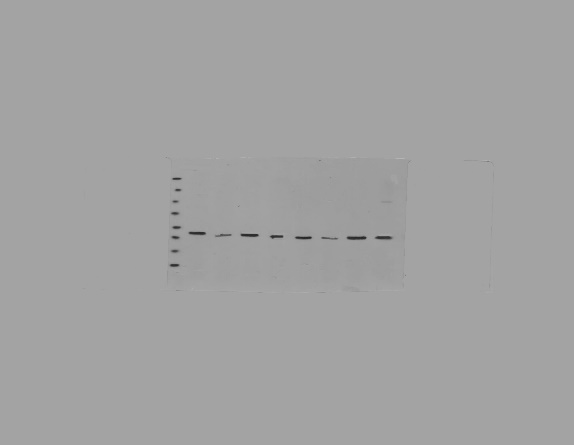 |
| --- | --- |
| 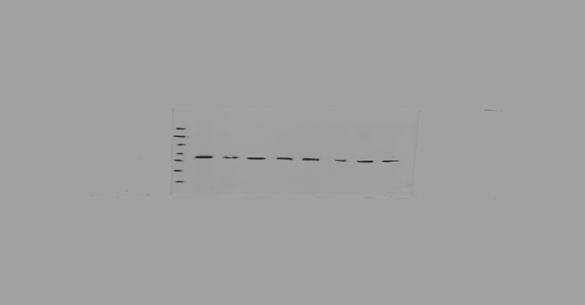 | |

AMPK

| 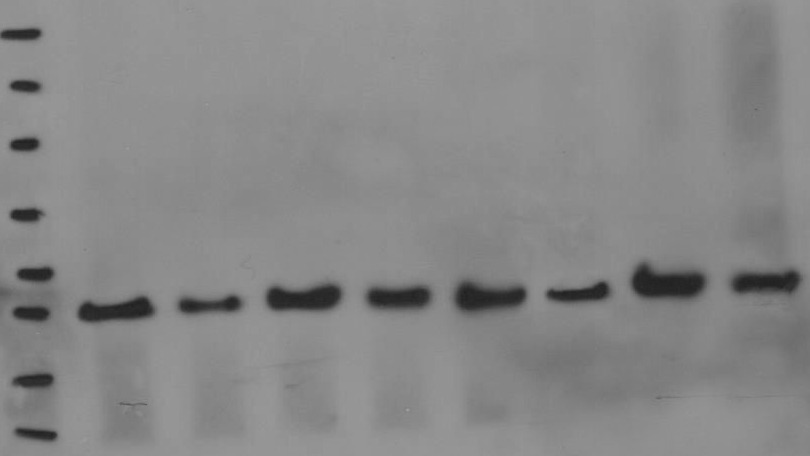 | 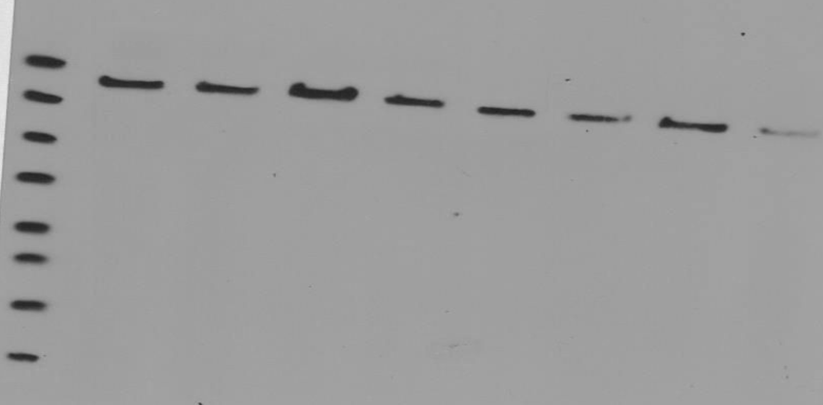 |
| --- | --- |
| 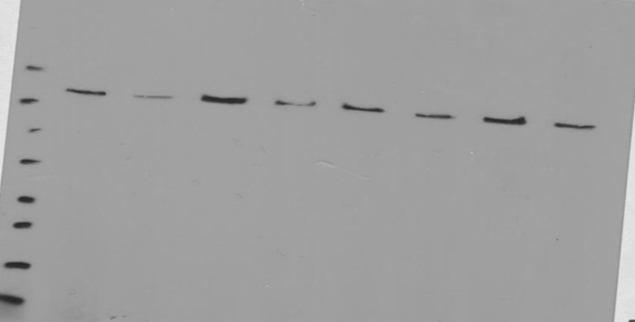 | |

ACC

| 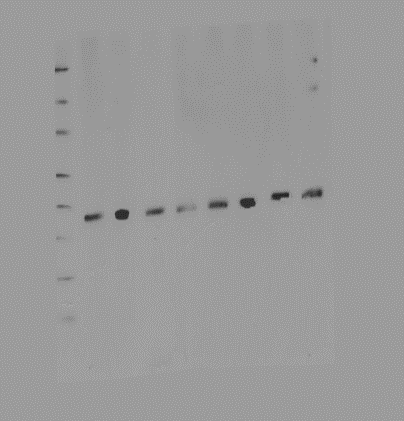 | 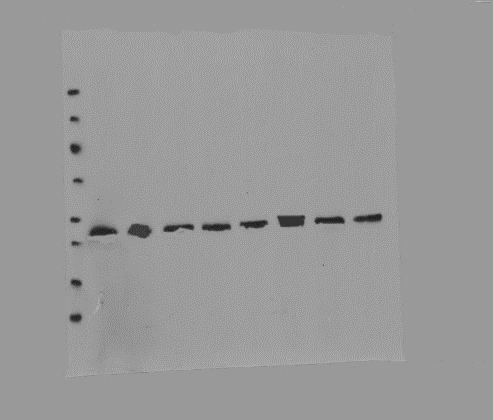 |
| --- | --- |
| 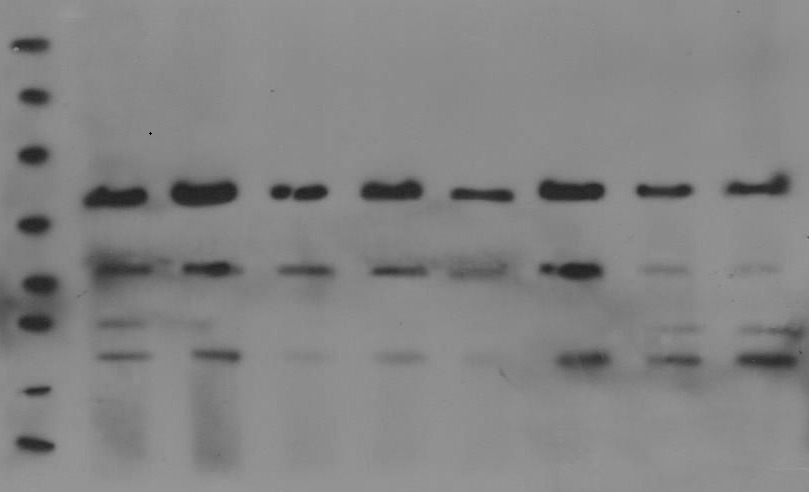 | |

SREBP-1c

| 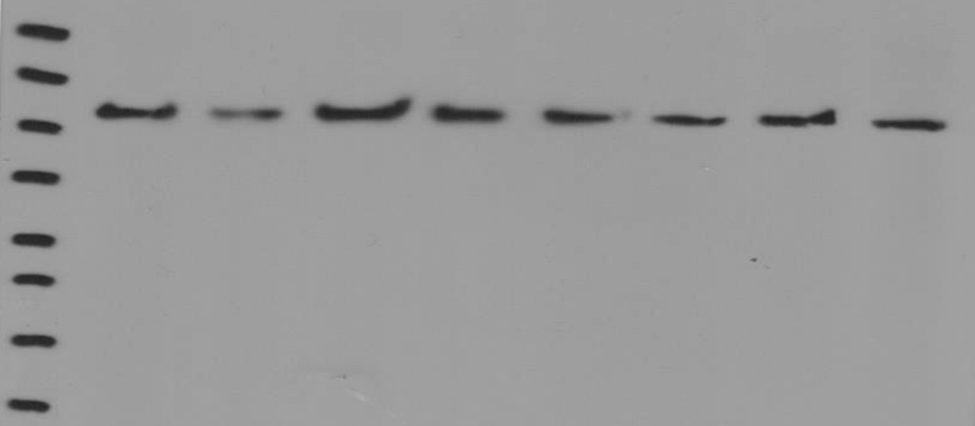 | 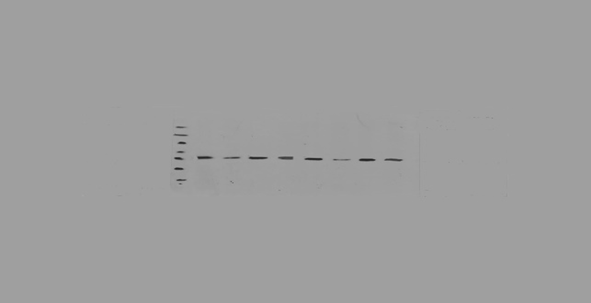 |
| --- | --- |
| 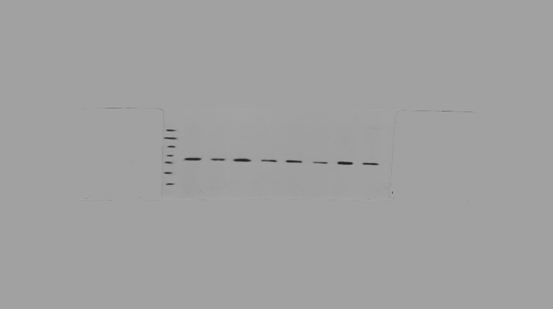 | |

FAS

| 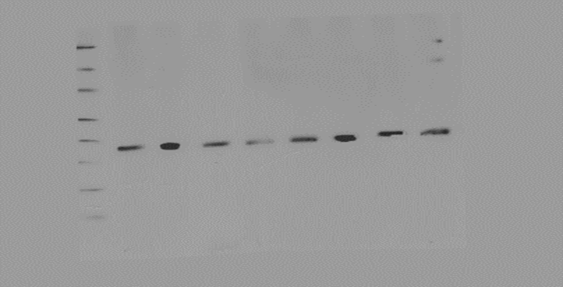 | 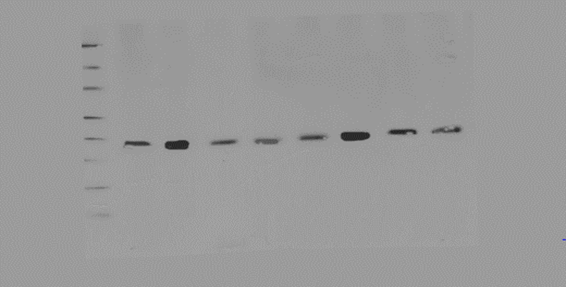 |
| --- | --- |
| 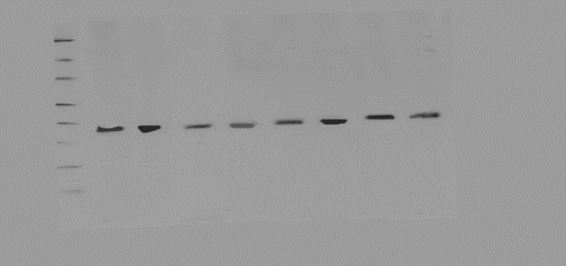 | |

β-Actine

| 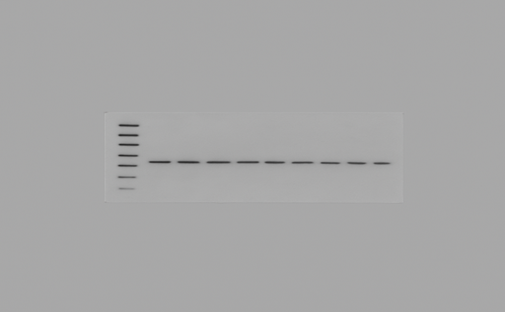 | 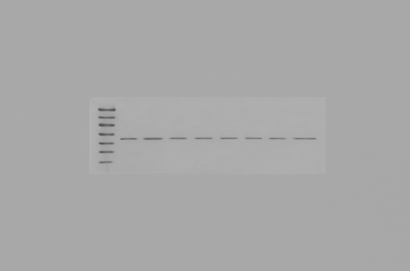 |
| --- | --- |
| 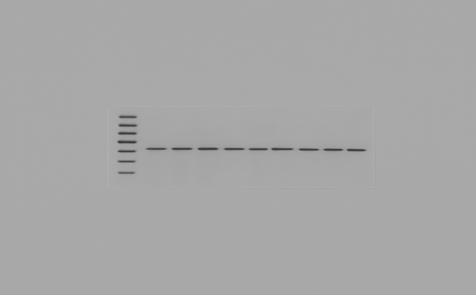 | |

Supplement: Supplementary file 1 — Supplementary Material 1 [file 41598_2025_15432_MOESM1_ESM.docx]
